# Disc Displacement of the Temporomandibular Joint and Facial Asymmetry in Children and Adolescents: A Systematic Review and Meta-Analysis

**DOI:** 10.3390/children9091297

**Published:** 2022-08-27

**Authors:** Oana Almășan, Daniel-Corneliu Leucuța, Smaranda Buduru

**Affiliations:** 1 Department of Prosthetic Dentistry and Dental Materials, Iuliu Hațieganu University of Medicine and Pharmacy, 32 Clinicilor Street, 400006 Cluj-Napoca, Romania; 2 Department of Medical Informatics and Biostatistics, Iuliu Hațieganu University of Medicine and Pharmacy, 400349 Cluj-Napoca, Romania

**Keywords:** jaw asymmetry, temporomandibular disorder, mandible, youths, minors

## Abstract

Subjects with facial skeletal asymmetries have a higher incidence of anterior temporomandibular joint disc displacement. The objective of the study was to consolidate existing evidence on the connection between temporomandibular joint disc displacement and mandibular asymmetry in youngsters and adolescents. A thorough examination was undertaken in the following databases: PubMed, Scopus, EMBASE, Web of Science, and Cochrane. To judge the publications’ methodological quality Newcastle Ottawa Scale was used. From the 1011 identified records, eight were selected for the qualitative synthesis and five for the quantitative synthesis, amounting to 692 subjects. Fifteen cephalometric variables were meta-analyzed. The distance from menton (Me) to midline (lateral mandibular asymmetry) was significantly shorter [−1.75 (95% CI −2.43–−1.07), *p* ≤ 0.001] in subjects with disc displacement compared to those without disc displacement. The distance from articulare (Ar) to gonion (Go) was significantly longer [3.74 (95% CI 1.04–6.44), *p* = 0.007] in subjects with disc displacement compared to those without disc displacement. The relationship between distance from articulare (Ar) to gonion (Go) or sella (S) to gonion (Go) and disc displacement was shown to be close to statistical significance level, but not for other cephalometric data. Disc displacement was associated with several cephalometric measurement variations in children and adolescents.

## 1. Introduction

Unilateral condylar bone changes were found to be linked with frontal craniofacial morphology [1]. Subjects with facial skeletal asymmetries have a higher incidence of temporomandibular disorders (TMDs) [2]. The menton shift was found to be significantly related to temporomandibular joint (TMJ) disc position on magnetic resonance imaging, with more deviation to the side with disc displacement [3]. It has been shown that unilateral anterior DD of the TMJ in adolescents can lead to mandibular asymmetry (MA), especially on the same side [4].

MA has been described as a contributing factor to temporomandibular disorders (TMDs) [5,6]. In young patients, mandible deviation and condylar bone changes have been associated with DD, with unilateral condylar bone changes causing mandible deviation on the same side [7]. Asymmetries in condylar movement and mandibular volume have been encountered in patients with MA, highlighting the close relationship between morphology and function [8]. A unilateral asymmetrically positioned mandible may result in asymmetrical condyles, especially on the affected side, due to the functional displacement of the mandible [9]. The development of MA may be connected to DD without reduction and changes in the mandibular condylar bone, with condylar modifications being more frequent on the deviated side [10]. In addition, it has been stated that on the side with the deviated mandible there was a higher probability of experiencing anterior DD [11]. The difference in condylar height between the unaffected and affected sides may increase the risk of MA, with the disc on the affected side shifting anteriorly [12]. It has been reported that jaw movements may be associated with craniofacial morphology, with the non-deviated side having a wider range of jaw movements than the deviated side [13]. It has been shown that TMD, unusual condyle modeling and craniofacial asymmetry are frequent and associated factors, with lengthened or wider condyles being observed on the shorter mandibular ramus side [14].

In subjects with juvenile idiopathic arthritis as a result of unilateral or asymmetrical TMJ involvement, limited mandibular movements have been encountered, mandibular deviation being associated with the affected side, which displayed the most severe facial asymmetry [15]. In patients with juvenile idiopathic arthritis, the asymmetry of the face, particularly around the chin, seemed to have been connected to asymmetrical TMJ destruction, however, the association between facial asymmetry and impacted TMJ is modest and underpowered [16].

As far as we are aware, there are currently no extensive studies to check the hypothesis that facial asymmetry or lateral mandible shift occurrence is similar in children and adolescents with and without TMJ disc displacements. Consequently, the study aimed at conducting a comprehensive review with a meta-analysis of existing studies on the relationship between TMJ DD and mandibular asymmetry in youths. 

## 2. Materials and Methods

### 2.1. Procedure and Enrollment

The systematic review was undertaken in accordance with the guidelines of the “Preferred Reporting Items for Systematic Reviews and Meta-Analyses Protocols (PRISMA) Statement” [17].

The Open Science Framework platform was used to register the study protocol prospectively on 25 July 2022 which can be found at the following location: https://osf.io/ax683 (accessed on 25 July 2022).

### 2.2. Standards for Selection

Original publications that explored the study goals, with a focus on the presence of facial asymmetry or lateral mandible shift in children or adolescents with TMJ DD, were the inclusion criteria. Subjects who had orthodontic or orthognathic therapy were excluded, as were systematic reviews, meta-analyses, scoping reviews, opinion pieces, comments, communications, cases, conference proceedings, editorials, and papers written in languages other than English.

### 2.3. Resources of Knowledge

In July 2022, an untimed organized electronic search was undertaken in the following databases: PubMed, Scopus, EMBASE, Web of Science, and Cochrane. Terms from MeSH and Emtree were utilized when suitable. On 20 July 2022, the final automated search of all databases was completed. Additionally, appropriate study reference lists were individually examined. On the Rayyan internet website, all citations were retrieved and sorted [18].

### 2.4. Methodology for Selection

There were no search filters or restrictions, nor was there a time limit on searches. The PECO framework served as the basis for the study design: Patient (P)-children or adolescents; Exposure (E)-with TMJ DD; Comparison (C)-without TMJ DD; Outcome (O)-facial anthropometric measurements or occurrence of facial asymmetry. A single search strategy was performed, that included the following terms: (“temporomandibular joint” OR “TMJ”) AND (“disk displacement” OR “disc displacement”) AND (“facial asymmetry” OR “lateral mandible shift” OR “lateral mandibular shift” OR “lateral mandible deviation” OR “lateral mandibular deviation”) AND “child”.

The complete search strategy adapted for the PubMed database is presented in Supplementary Table S1.

### 2.5. Recruitment Procedure

Rayyan was used to eliminate redundancies from the output lists of results from all databases. The papers were organized, and an objective, blind assessment of the included papers was conducted. The “blind on” mode was used to decrease selection bias. The residual findings were exported to an Excel spreadsheet which was provided as a digital format for screening, retrieval, and quality evaluation. (Microsoft Office 365, MS, Redmond, WA, USA). Zotero software version 6.0.6 was used to handle all citations [19]. Two researchers (O.A., D.C.L.) separately examined the eligible studies and corroborated if the item should be included. The chosen publications were acquired in full text and individually appraised, with disagreements addressed through negotiation. The rationale for each excluded item was documented.

### 2.6. Technique for Data Gathering

**Figure 1:**
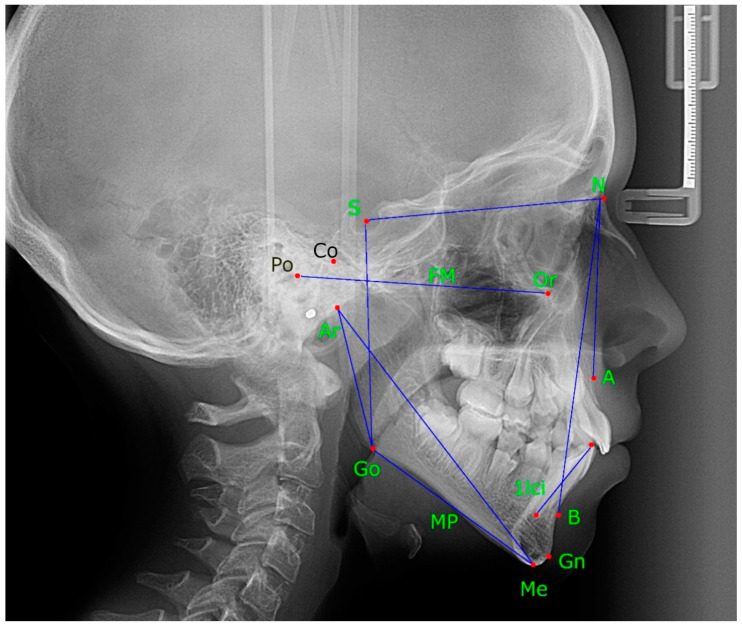
Lateral cephalogram–anthropometric landmarks and lines. S, sella; N, nasion; SNA, the angle between sella, nasion and point A; SNB, the angle between sella, nasion and point B; ANB, the angle between point A, and point B; Go, gonion; Co, condylion; Ar, articulare; Gn, gnathion; Po, porion; Me, menton; 1lci, 1 lower central incisor; NB, nasion point B line; FM, Frankfurt plane; MP, mandibular plane.

**Figure 2:**
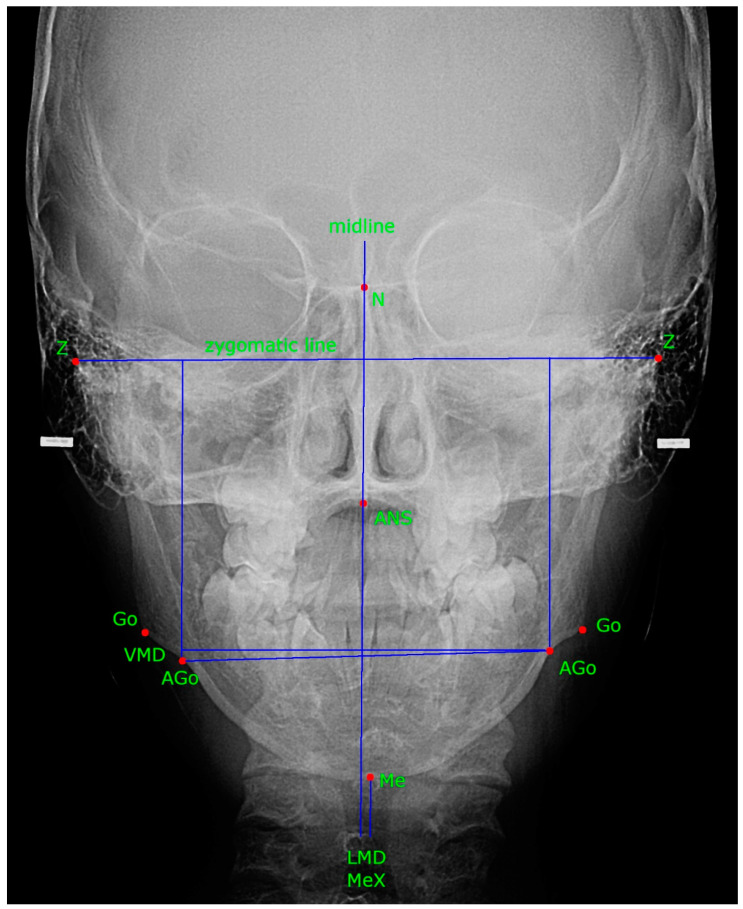
Posteroanterior cephalogram–anthropometric landmarks and lines. Go, gonion; AGo, antegonion; N, nasion; Z, zygomatic point; ANS, anterior nasal spine; Me, menton, VMD, vertical mandibular displacement; LMD, lateral mandibular displacement; MeX, menton to the midline.

In the uniform Microsoft Excel sheet file, two researchers retrieved data from the publications, following parameters were recorded: author names and publishing year, name of publication, summary, keywords, study objective, study population, DD classification, asymmetry, radiographic evaluation, findings (facial anthropometric measurements—Figure 1 and Figure 2), conclusions.

### 2.7. Critical Evaluation of Each Study

The quality of evidence of the qualifying papers included in our research was assessed using the Newcastle Ottawa Scale: checklist for observational case-control publications [20]. We considered the presence of DD as defining the case group and the absence of otherwise or similar groupings regarding DD presence in unilateral or bilateral situations. The cephalometric variables were considered as the exposure. 

### 2.8. Synthesis Methods

Since the results of different articles in the meta-analysis offered either the statistics of two compared groups or the difference between the two groups, we chose to compute the effect size as the difference between the DD and normal disc (ND) position groups, along with the standard error (SE). Where the standard deviation (SD) remained unavailable, it was determined by employing confidence intervals (CIs) or *p*-values, according to the Cochrane Handbook criteria [21]. On the computed mean differences and SE, meta-analyses were conducted using the meta program [22]. Because of variability among investigations, the random effects model was employed to calculate the conventional difference between the means and ninety-five percent confidence interval (CI) for each variable. To examine statistical variance between trials, the chi-square Q-test and I2 were implemented. An analysis with one variable removed was performed to see how reliable the results were. The assumption of statistical significance was made if the *p*-value was less than 0.05. The R environment for statistical computation and visualization (R Foundation for Statistical Computing, Vienna, Austria) version 4.1.2 was used to perform the calculations [23].

### 2.9. Identification of Bias

The risk of bias could not be established due to the small number of papers. Therefore, we chose the Egger test to investigate articles’ bias.

## 3. Results

### 3.1. Selection of Sources of Evidence

**Figure 3:**
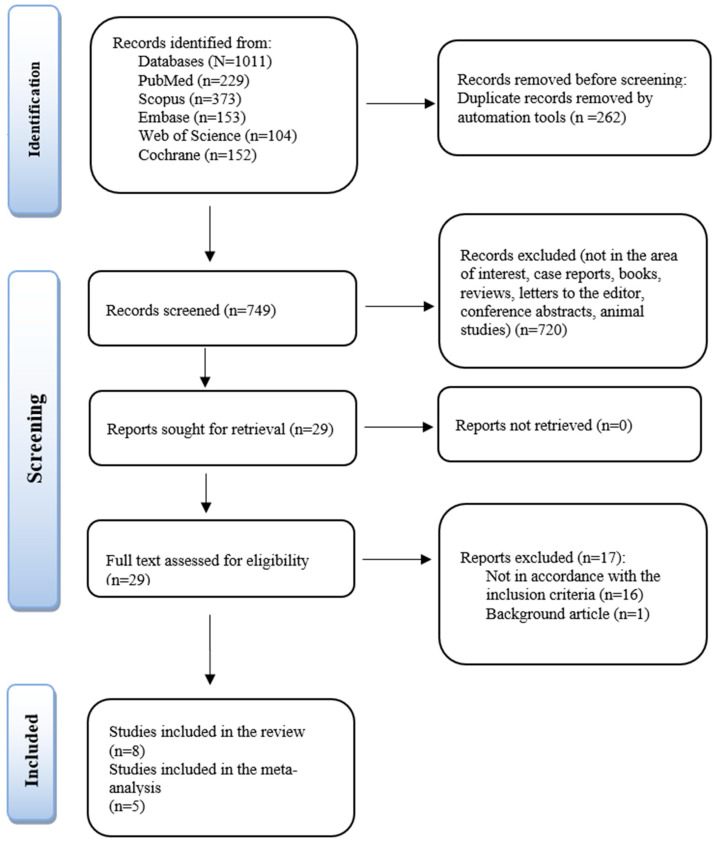
PRISMA illustration of the chosen studies.

A total of 1011 were enrolled after applying the search strategy (229 via PubMed; 373 of Scopus; 153 of EMBASE; 104 of Web of Science and 152 via Cochrane). Following the removal of similar documents, a number of 749 papers were screened. The included studies were chosen during the initial phase based on their title, abstract, and relation to the research question. The remaining 29 articles’ full texts were obtained and reviewed for eligibility. After reading all the publications that were considered for eligibility, eight have been included in the review, of which five were employed in the meta-analysis. A PRISMA flowchart serves to illustrate the recruiting and screening process (Figure 3).

### 3.2. Features of Research

Table 1 summarizes the features of the selected investigations, as well as the (1) authorship and date, (2) study aim, (3) study population, (4) disc displacement classification, (5) asymmetry, (6) radiographic evaluation, (7) author’s findings, and (8) author’s conclusion. In the eight studies included in the qualitative synthesis, there were 692 subjects involved. In the five studies from the quantitative synthesis, a total number of 515 subjects were included. Seven studies used the MRI method for disc position classification, whereas just one study used temporomandibular disorders investigation criteria for a diagnosis (RDC/TMD) [24]. Radiographic evaluation used lateral cephalogram in four studies [24,25,26,27], and posteroanterior cephalogram in five studies [3,4,26,28,29], Trpkova et al. [26] used both methods. Bilateral DD was reported in four studies [3,25,26,28], unilateral DD was reported in [3,4,26,28,29], while two studies did not report the presence of either unilateral or bilateral DD [24,27].

### 3.3. Results of Syntheses

#### 3.3.1. Distance from Menton to Midline (Mandibular Lateral Asymmetry, or Displacement)

**Figure 4:**
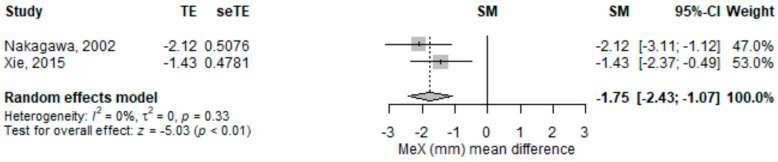
Forest plot for (mm) standardized mean change difference. MeX−distance from menton to midline; TE−effect; seTE—effect’s standard error; SM−the average discrepancy; CI-interval of confidence.

The distance from menton to midline (in mm) on the posteroanterior cephalogram was significantly lower [−1.75 (95% CI −2.43–−1.07), *p* ≤ 0.001] in subjects with disc displacement compared to those without disc displacement in the random-effects meta-analysis model (Figure 4). The heterogeneity was low (I2 = 0%) and not statistically significant. Both studies had statistically significant results, pointing in the same direction. The results are robust to leave-one-out sensitivity analyses.

#### 3.3.2. Distance from Articulare to Gonion (Mandible Ramus Height)

**Figure 5:**
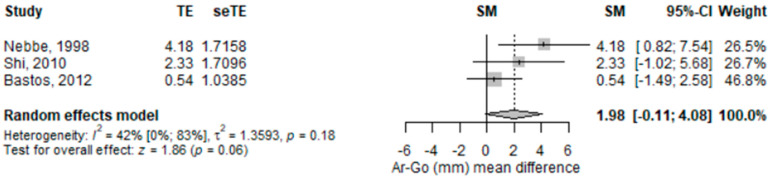
Forest plot for distance from articulare to gonion (mm) standardized mean change difference. Ar-Go—distance from articulare to gonion; TE—effect; seTE—effect’s standard error the effect; SM—the average discrepancy; CI—confidence interval.

The distance from articulare to gonion, (in mm) on the lateral cephalogram was higher [1.98 (95% CI −0.11–4.08), *p* = 0.063] in subjects with disc displacement compared to those without disc displacement in the random-effects meta-analysis model (Figure 5), but it did not reach the significance threshold. The heterogeneity was moderate (I2 = 42.3%), albeit not statistically significant. Only one study out of the three included had a statistically significant result pointing in the same direction. Omitting Bastos study [24] in the leave-one-out sensitivity analyses modified the pooled result to be significant, but the exclusion of any of the other studies did not have the same effect (Supplementary Figure S8).

#### 3.3.3. Distance from Articulare to Menton (Total Mandibular Length)

**Figure 6:**
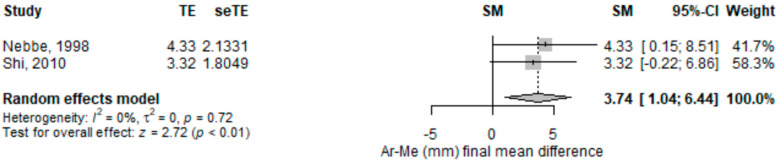
Forest plot for distance from articulare to menton (mm) standardized mean change difference. Ar-Me—distance from articulare to menton; TE—effect; seTE—effect’s standard error of the effect; SM—the average discrepancy; CI—confidence interval.

The distance from articulare to menton (in mm) on the lateral cephalogram was significantly higher [3.74 (95% CI 1.04–6.44), *p* = 0.007] in subjects with disc displacement compared to those without disc displacement in the random-effects meta-analysis model (Figure 6). The heterogeneity was low (I2 = 0%), and not statistically significant. Only one study out of the two included had a statistically significant result, and both pointed in the same direction. The other study, Shi was close to statistical significance [27].

#### 3.3.4. Distance from Sella to Gonion (Overall Posterior Jawline Dimension)

**Figure 7:**
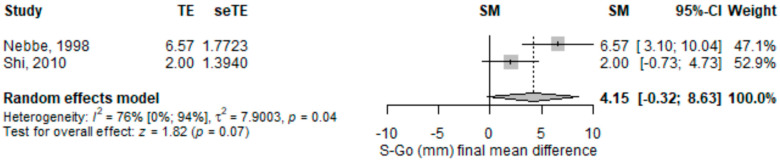
Forest plot for distance from sella to gonion (mm) standardized mean change difference. S-Go—distance from sella to gonion; TE TE—effect; seTE—effect’s standard error; SM—the average discrepancy; CI—confidence interval.

The length between sella to gonion (in mm) on the lateral cephalogram was significantly higher [4.15 (95% CI −0.32–8.63), *p* = 0.069] in subjects with disc displacement compared to those without disc displacement in the random-effects meta-analysis model (Figure 7). The heterogeneity was substantial (I2 = 76%), and statistically significant. Only one study out of the two included had a statistically significant result, and both pointed in the same direction.

#### 3.3.5. Other Cephalogram Measurements

The other cephalogram measurements were not statistically significant (Table 2, Supplementary Figures S1–S7).

### 3.4. Investigation’s Potential Bias

The Newcastle Ottawa Scale (NOS) was engaged to evaluate the scientific research (Table 3). The case and control definitions were adequately followed in the majority of the studies since they used MRI to diagnose the disc position, except for one study that used research diagnostic criteria for temporomandibular disorders [24]. The case representativeness was reported in only two studies that either used a consecutive sample [4] or all the subjects in their database [3]. But all the other studies did not specify if all the subjects were selected from the same source. The comparability of the groups was partially assured by five studies since they studied only female subjects [25,26,27,28,29]. Only one study matched the groups by gender, Angle’s categorization of misaligned teeth, and the cervical vertebra development score [24]. All the studies can be considered to have an appropriate exposure ascertainment by using cephalometric measurements.

## 4. Discussion

### 4.1. Scientific Proof Synopsis

The present study showed a significantly lower distance from menton to midline (mandibular lateral displacement), measured on posteroanterior cephalogram, and a higher distance from articulare to gonion (mandible ramus height) measured on the lateral cephalogram in subjects with disc displacement compared to those with normal disc position; furthermore, for the relation between the distance from articulare to gonion (the height of the mandibular ramus) or from sella to gonion (total posterior facial height) and disc displacement, the results were near the significance level, but not for other cephalometric measurements.

Mandibular asymmetry was evaluated on posteroanterior cephalograms by the distance of the menton to the midline (mandibular lateral displacement, or asymmetry) in [3,4,28,29]. Another way to indicate the mandibular asymmetry was the vertical mandibular displacement, as the distance between antegonion and the zygomatic arch line (mandibular height) [28]. Trpkova et al. [26] used a formula to calculate the asymmetry between the right and left side for different cephalometric measurements: (right − left)/(right + left)/200. Xie et al. [4], had no control group but used a longitudinal self-control design to assess whether unilateral anterior DD would lead to asymmetry of the mandible or of the mandible condyle, the mean follow-up being 12.2 months. The study observed that unilateral juvenile anterior DD leads in time to shorter condylar height on the same side and MA. Bastos [24] divided the study and the control group depending on the cervical spine development Bastos et al., identified a connection between the TMJ state and a hyperdivergent face growth pattern in youths [24]. The onset of the DD was found to be related to the mandibular DD, by Nakagawa [28]. The menton deviation was significantly correlated with the disc position, being more deviated to the more affected side, and related to the unilateral as well the bilateral DD [3]. Young girls with incomplete disc displacement and Class II, Division 1 dentition may show transverse but not longitudinal abnormalities in the jaw [27].

MA has also been reported to be much more widespread and extensive in young patients with unilateral DD, with the degree of asymmetry being linked with condyle height and disc morphology [29]. Patients with DD had a shorter jaw length as well as a backward jaw position, suggesting that DD is linked with abnormal structural architecture [30]. According to research, it has been shown that there is a clear relationship between severe DD and skeletal deformities in orthodontic patients [31]. DD affects facial morphology, the differences becoming more pronounced with the progress of the displacement, highlighting the significance of early DD diagnosis and treatment [32].

To encourage temporomandibular condyle natural growth and prevent facial deformity, DD in young individuals should be corrected as soon as feasible, especially if it is asymmetric [33]. In young patients with unilateral anterior DD, arthroscopic disc repositioning has been shown to improve facial growth [33].

### 4.2. Strengths and Weaknesses

The papers considered in this study had several drawbacks. The most frequently encountered issue was the representativeness of the cases that were not reported, as well as the diversity of clinical settings that generated the study cohorts. The other problems relating to the quality of the articles were the use of RDC/TMD instead of MRI for the DD diagnosis and the absence of measures to aid comparability–but luckily, only one study for each problem had this issue. Being cross-over studies, the causality between DD and facial asymmetry cannot be augmented. Nevertheless, a strong association was observed for several cephalometric variables. Furthermore, one study observed in a prospective cohort of children the increase of facial asymmetry with time and in relation to DD. For sure, the question of who the cause is will remain debatable.

In addition, our evaluation includes the following strengths: this is the first holistic research and meta-analysis of facial asymmetry in youths; both posteroanterior and lateral cephalogram measurements were assessed; a thorough search approach was employed; considerable representative databases were explored (PubMed, Scopus, EMBASE, Web of Science, and Cochrane); sensitivity analyses were usedand fifteen cephalometric variables were meta-analyzed.

## 5. Conclusions

In patients with disc displacement compared to those with normal disc position, the present study identified a significantly reduced distance from menton to midline on the posteroanterior cephalogram and a larger distance from articulare to gonion on the lateral cephalogram.

## Figures and Tables

**Table 1:** Study characteristics.

Author, Year	Aim	Study Population	DD Classification	Asymmetry	Radiographic Evaluation	Findings	Conclusions
Nebbe, 1998 [25]	to test the hypothesis of no difference in facial cephalometric measurements in adolescents with DD	study group: bilateral DD 17 teenage girls, 13.65 years on average Control group: ND position 17 teenage girls, 13.53 years on average	MRI	study group: lower overall posterior height (S-Go) reduced mandibular ramus length (Co-Go, Ar-Go) shortened posterior facial height (S-Ar) increase in the distance from S-N to the palatal plane increase in the distance from S-N to a line tangent to the inferior border of the mandible’s body posterior displacement of Gn related to anterior structures of the face posterior rotation of the mandible Control group: higher mandibular lateral displacement inclined frontal occlusal plane	lateral cephalogram	mean differences (95% CI): Ar-Go (mm) 4.18 (0.69–7.68); Ar-Me 4.33 (mm) (−0.01–8.68); Ar-Go-Me (degrees) 0.46 (−4.18–3.27); FH/MP 3.80 (degrees) (−8.02–0.42); S-Go (mm) 5.57 (1.96–9.18); N-Me (mm) 1.07 (−5.54–3.40)	bilateral DD: posterior vertical facial height diminished; Juvenile disc position aberrations are not within the range of typical physiologic diversity.
Trpkova, 2000 [26]	if TMJ ID (DD) is associated with craniofacial asymmetry	80 females (average age, 13.20 ± 1.7) bilateral normal TMJ: 42 unilateral right TMJ ID: 13 unilateral left TMJ ID: 10 bilateral TMJ ID: 15	MRI TMJ ID: disc displacement and disc length	the longitudinal imbalance in the area of AGo differed substantially	posteroanterior cephalograms lateral cephalograms	increased asymmetry of the AGo with a shorter mandible ramus in bilateral TMJ ID	women with symmetrical TMJ ID experienced higher vertical mandible asymmetry
Nakagawa, 2002 [28]	to determine the relationship between LMD, VMD, DD, and mandible growth	54 female adolescents (average age: 15.7 ± 3.0) Group 1: bilateral ND position: 23 subjects (average age: 14.9 ± 3.4); Group 2: unilateral /bilateral DD (=partial DD): 12 subjects (average age: 15.9 ± 2.9) Group 3: unilateral /bilateral disc dislocation (=complete DD): 19 subjects (mean age: 16.4 ± 2.4 years)	MRI	right and left mandibular height (VMD) LMD	posteroanterior cephalograms	mandible deviation is linked to DD and disc dislocation Group 1: VMD (AGo-zygomatic line): mean 0.89 ± 0.74 mm LMD (MeX) mean 1.33 ± 1.23 mm Group 2: VMD mean 3.2 ± 1.51 mm LMD mean 3.01 ± 2.51 mm Group 3: VMD mean 3.13 ± 2.3 mm LMD mean 3.72 ± 2.42 mm	DD disturbs normal mandible growth VMD was not related to age LMD was related to age DD was related to LMD and VMD
Shi, 2010 [27]	to evaluate the relationship between partial DD and mandibular dysplasia	46 female adolescents aged 10.1–12.8 years. DD group (*n* = 26), ND group (*n* = 20)	MRI	the displaced group exhibited a reduced length of the mandible (Go-Po), sharper mandible plane (MP/FH), and steep mandible inclination (Ar-Go-Me)	lateral cephalograms	DD vs. ND: SNA(°) 79.31 ± 3.40 vs. 80.15 ± 4.79, *p* = 0.489; SNB(°) 74.31 3.06 vs. 75.25 5.09, *p* = 0.440; ANB(°) 6 ± 1.45 vs. 6 ± 1.02, *p* = 1; Ar-Go(mm) 45.42 ± 4.59 vs. 47.75 ± 6.50, *p* = 0.162; Ar-Me(mm) 95.73 ± 4.68 vs. 99.05 ± 6.95, *p* = 0.060; Go-Po(mm) 69.00 ± 3.96 vs. 72.00 ± 3.54, *p* = 0.011 *; Ar-Go-Me (°) 118.77 ± 5.03 vs. 115.75 ± 2.78, *p* = 0.020 *; MP/FH (°) 31.23 ± 3.85 vs. 26.80 ± 5.54, *p* = 0.003; S-Go(mm) 74.50 ± 3.26 vs. 76.50 ± 5.57, *p* = 0.134; N-Me(mm) 116.12 ± 4.22 vs. 116.30 ± 4.96, *p* = 0.892	partial DD may be related to horizontal jaw impairments but not longitudinal abnormalities
Bastos, 2012 [24]	to evaluate differences between the cephalometric variables for facial growth patterns in children and adolescents with articular TMD and control group	Experimental group 30 patients with articular TMD. Control group: 30 volunteers without TMD, matched	RDC/ TMD	the analysis of the post-peak of pubertal growth spurt showed that the experimental group had mean values for SNA and SNB angles decreased, and the facial axis angle (SN.Gn) and lower incisor inclination (1-NB) increased with the mean values found in the control group, revealing statistically significant differences	lateral cephalograms	DD vs. ND:Pre-peak: S.N.A (°) 82.05 ± 3.03 vs. 81.39 ± 4.34, *p* = 0.611; S.N.B (°) 4.30 ± 1.91 vs. 3.98 ± 4.63, *p* = 0.799; A.N.B (°) 4.3 ± 1.91 vs. 3.98 ± 4.63, *p* = 0.799; Ar-Go (mm) 39.22 ± 3.86 vs. 39.46 ± 3.50, *p* = 0.853; Ar.Go.Me (°) 129.66 ± 6.75 vs. 126.15 ± 5.37, *p* = 0.104; S-Go (mm) 68.25 ± 6.10 vs. 67.23 ± 5.68, *p* = 0.619; N-Me (mm) 110.40 ± 7.96 vs. 110.24 ± 7.69, *p* = 0.952; post-peak: S.N.A (°) 78.25 ± 3.55 vs. 82.90 ± 4.53, *p* = 0.008; S.N.B (°) 74.69 ± 3.63 vs. 79.26 ± 4.75, *p* = 0.011; A.N.B (°) 3.55 ± 2.98 vs. 3.71 ± 2.89, *p* = 0.891; Ar-Go (mm) 42.18 ± 3.53 vs. 43.12 ± 3.99, *p* = 0.532; Ar.Go.Me (°) 125.76 ± 5.59 vs. 128.02 ± 4.42, *p* = 0.265; S-Go (mm) 73.42 ± 6.42 vs. 72.99 ± 4.36, *p* = 0.842; N-Me (mm) 121.26 ± 9.21 vs. 115.37 ± 7.58, *p* = 0.088	changes in morphometric parameters were detected in youngsters with joint TMD
Xie, 2015 [29]	to determine the amount of MA in asymmetric ADD individuals	study goup: average age 16.74 years vs. average age 16.21 years in the control group (165 patients with ADD (101 left, 64 right), 156 controls without ADD	MRI	of 119 MA patients in ADD group, 73 with left ADD, 46 with right ADD,	posteroanterior cephalograms	in the ADD group, category 27.88% had no MA, mean MeX: 5.62 mm in the control group, 25.64% had MA, mean MeX: 4.19 mm	MA is more unilateral ADD teenagers The greater the DD, the shorter the condyle and higher the jaw irregularity
Xie, 2016 [4]	to observe the influence of ADD and to analyze its effect on the symmetry of the mandible	average age 16.31 28 females, 16 males The average follow-up period was 12.22 months	MRI	first evaluation 86.36% MA follow-up: 93.18% MA	posteroanterior cephalograms	the correlation coefficient between condyle height disparity and MeX (CC = 0.681, *p* < 0.05) the increase of menton deviation was significantly related to the age of patients at the initial visit (correlation coefficient = −0.760, *p* < 0.05). the average MeX was 5.58 mm at the initial visit, while it was 7.74 mm after follow-up	in adolescents, MA was secondary or fostered by UJADD
Guercio-Monaco, 2020 [3]	to analyze the association between TMJ disc position evaluated by MRI and the mandible deviation evaluated by PA in adolescents	53 adolescents (37 females and 16 males, mean age 14.28 ± 2.46 years; 11–18) and 106 TMJs group I Same disc position bilateral (*n* = 23); group II DD is more severe ipsilateral (*n* = 17); group III DD more severe contralateral (*n* = 13)	MRI	significant differences between the mean of group II (4.4 ± 2.2) with groups I and III (*p* = 0.016 and *p* = 0.036 respectively), with a greater menton deviation concerning the rest of the groups a statistical association between DD and gender was observed (*p* = 0.002), with more frequent DD in females	posteroanterior cephalograms	MeX menton deviation: Same disc position bilateral 2.17 ± 1.93; DD more severe ipsilateral 4.40 ± 2.26; DD more severe contralateral 2.10 ± 1.70	the menton deviation was related to unilateral or bilateral cases TMJ DD the menton tended to exhibit more deflection to the side more affected

DD, disc displacement; ND, normal position of the articular disc; ADD, disc displacement towards the anterior; UJADD, unilateral juvenile anterior disc displacement; S, Sella; SNA, the angle between sella, nasion and point A; SNB, the angle between sella, nasion and point B; ANB, the angle between point A, nasion and point B; Go, gonion; Co, condylion; Ar, articulare; N, nasion; Gn, gnathion; AGo, antegonion; Po, porion; Me, menton; 1, lower central incisor; NB, nasion point B line; FM, Frankfurt plane; MP, mandibular plane; MA, mandibular asymmetry; TMJ, temporomandibular joint; TMD, temporomandibular joint disorder; RDC, research diagnostic criteria; ID, internal derangement; MRI, magnetic resonance images; LMD, lateral mandibular displacement; VMD, vertical mandibular displacement; MA, mandibular asymmetry; MeX, menton to midline. *, statistically significant.

**Table 2:** Meta-analyses results.

Characteristic, Effect Size Type	Number of Studies	Effect Size (95% CI)	*p*-Value	I2 (95% CI)	*p*-Value	Egger Test	Studies	Leave One Out
MeX (mm) mean difference	2	−1.75 (−2.43–−1.07)	<0.001	NC		NC	Nakagawa, 2002 [28]; Xie, 2015 [29]	-Nakagawa, 2002: −1.43 (−2.37–−0.49), *p* = 0.003, I2 = NA%; -Xie, 2015: −2.12 (−3.11–−1.12), *p* ≤ 0.001, I2 = NA%
Ar-Go (mm) mean difference	3	1.98 (−0.11–4.08)	0.063	42.3 (0–82.6)	0.177	0.265	Nebbe, 1998 [25]; Shi, 2010 [27]; Bastos, 2012 [24]	-Nebbe, 1998: 1.02 (−0.71–2.76), *p* = 0.248, I2 = 0%; -Shi, 2010: 2.11 (−1.42–5.63), *p* = 0.242, I2 = 70%; -Bastos, 201: 3.25 (0.88–5.63), *p* = 0.007, I2 = 0%
Go-Po (mm) mean difference	2	1.3 (−2.37–4.97)	0.487	NC		NC	Shi, 2010 [27]; Bastos, 2012 [24]	-Shi, 2010: −0.76 (−3.93–2.41), *p* = 0.636, I2 = NA%; -Bastos, 2: 3 (0.83–5.17), *p* = 0.007, I2 = NA%
Ar-Me (mm) mean difference	2	3.74 (1.04–6.44)	0.007	NC		NC	Nebbe, 1998 [25]; Shi, 2010 [27]	-Nebbe, 1998: 3.32 (−0.22–6.86), *p* = 0.066, I2 = NA%; -Shi, 2010: 4.33 (0.15–8.51), *p* = 0.042, I2 = NA%
S-Go (mm) mean difference	2	4.15 (−0.32–8.63)	0.069	NC		NC	Nebbe, 1998 [25]; Shi, 2010 [27]	-Nebbe, 1998: 2 (−0.73–4.73), *p* = 0.151, I2 = NA%; -Shi, 2010: 6.57 (3.1–10.04), *p* ≤ 0.001, I2 = NA%
N-Me (mm) mean difference	3	−0.19 (−2.24–1.86)	0.859	0 (0–89.6)	0.471	0.721	Nebbe, 1998 [25]; Shi, 2010 [27]; Bastos, 2012 [24]	-Nebbe, 1998: −0.61 (−3.09–1.87), *p* = 0.631, I2 = 8%; -Shi, 2010: −0.7 (−4.34–2.93), *p* = 0.705, I2 = 26%; -Bastos, 201: 0.43 (−1.86–2.73), *p* = 0.711, I2 = 0% Supplementary Figure S10
SNA (deg) mean difference	2	1.31 (−0.28–2.9)	0.105	NC		NC	Shi, 2010 [27]; Bastos, 2012 [24]	-Shi, 2010: 1.64 (−0.43–3.71), *p* = 0.12, I2 = NA%; -Bastos, 2: 0.84 (−1.63–3.31), *p* = 0.506, I2 = NA%
SNB (deg) mean difference	2	2.82 (−0.74–6.37)	0.12	NC		NC	Shi, 2010 [27]; Bastos, 2012 [24]	-Shi, 2010: 4.57 (2.43–6.71), *p* ≤ 0.001, I2 = NA%; -Bastos, 2: 0.94 (−1.58–3.46), *p* = 0.465, I2 = NA%
ANB (deg) mean difference	2	−0.02 (−0.67–0.64)	0.958	NC		NC	Shi, 2010 [27]; Bastos, 2012 [24]	-Shi, 2010: −0.11 (−1.76–1.53), *p* = 0.894, I2 = NA%; -Bastos, 2: 0 (−0.71–0.71), *p* = 1, I2 = NA%
MP/FH (deg) mean difference	2	−0.45 (−8.51–7.61)	0.913	NC		NC	Nebbe, 1998 [25]; Shi, 2010 [27]	-Nebbe, 1998: −4.43 (−7.27–−1.59), *p* = 0.002, I2 = NA%; -Shi, 2010: 3.8 (−0.26–7.86), *p* = 0.067, I2 = NA%
Ar-Go-Me (deg) mean difference	3	−1.55 (−3.52–0.41)	0.121	30.8 (0–92.8)	0.236	0.06	Nebbe, 1998 [25]; Shi, 2010 [27]; Bastos, 2012 [24]	-Nebbe, 1998: −2.23 (−4.15–−0.31), *p* = 0.023, I2 = 11%; -Shi, 2010: −0.42 (−2.69–1.84), *p* = 0.715, I2 = 0%; -Bastos, 201: −1.57 (−4.93–1.8), *p* = 0.362, I2 = 61% Supplementary Figure S9

S, Sella; SNA, the angle between sella, nasion, and point A; SNB, the angle between sella, nasion, and point B; ANB, the angle between point A, nasion, and point B; Go, gonion; Co, condylion; Ar, articulare; N, nasion; Po, porion; Me, menton; MP, mandibular plane; FM, Frankfurt plane; MA, mandibular asymmetry; MeX, menton to midline; CI, confidence interval; NC—cannot be computed due to a low number of studies.

**Table 3:** Newcastle Ottawa Scale rating of the identified papers.

Researcher and Release Year	Case Definition Sustainability	Cases’ Representativeness	Controls Selecting	Controls Defining	Cases and Controls Comparability	Exposure Assessment
Nebbe, 1998 [25]	*		*	*	*	*
Trpkova, 2000 [26]	*		*	*	*	*
Nakagawa, 2002 [28]	*		*	*	*	*
Shi, 2010 [27]	*		*	*	*	*
Bastos, 2012 [24]			*		**	*
Xie, 2015 [29]	*		*	*	*	*
Xie, 2016 [4]	*	*	*	*	NA	*
Guercio-Monaco, 2020 [3]	*	*	*	*		*

NA—not applicable.

## Data Availability

Data is provided in the paper.

## Supplementary Materials

The following supporting information can be downloaded at: https://www.mdpi.com/article/10.3390/children9091297/s1, Table S1: Search strategies for PubMed database. Figure S1: Forest plot for (deg) standardized mean change difference. SNA—sella nasion point A angle, TE—effect; seTE—the standard error of the effect; SM—mean difference; CI—confidence interval.; Figure S2: Forest plot for (deg) standardized mean change difference. SNB-sella nasion point B angle, TE—effect; seTE—the standard error of the effect; SM—mean difference; CI—confidence interval.; Figure S3: Forest plot for (deg) standardized mean change difference. ANB-point A—nasion-point B angle, TE—effect; seTE—the standard error of the effect; SM—mean difference; CI—confidence interval.; Figure S4: Forest plot for (deg) standardized mean change difference. MP—mandibular plane, FM-Frankfurt plane, TE—effect; seTE—the standard error of the effect; SM—mean difference; CI—confidence interval.; Figure S5: Forest plot for (deg) standardized mean change difference. Ar-articulare, Go-gonion, Me-menton, TE—effect; seTE—the standard error of the effect; SM—mean difference; CI—confidence interval.; Figure S6: Forest plot for (mm) standardized mean change difference. Go-gonion, Po = porion, TE—effect; seTE—the standard error of the effect; SM—mean difference; CI—confidence interval.; Figure S7: Forest plot for (mm) standardized mean change difference. TE—effect; seTE—the standard error of the effect; SM—mean difference; CI—confidence interval.; Figure S8: Leave-one-out sensitivity analysis for articulare to gonion distance Θ ^ = mean difference; Figure S9: Leave-one-out sensitivity analysis for Ar-Go-Me Θ ^ = mean difference, Ar-articulare, Go-gonion, Me-menton; Figure S10: Leave-one-out sensitivity analysis for N-Me Θ ^ = mean difference, N-nasion, Me-menton.
