# Supplementary material for: Disc Displacement of the Temporomandibular Joint and Facial Asymmetry in Children and Adolescents: A Systematic Review and Meta-Analysis
Source: Children (Basel). 2022 Aug 27;9(9):1297. doi: 10.3390/children9091297 (PMC9497886; doi:10.3390/children9091297)
Supplement: Supplementary file 1 [file children-09-01297-s001.zip › children-1860410-supplementary.pdf]

Supplementary file

Disc Displacement of the Temporomandibular Joint and Facial Asymmetry in Children and Adolescents: A Systematic Review and Meta-Analysis.

Journal: Children

Authors: Oana Almășan, Daniel-Corneliu Leucuța, Smaranda Buduru

Funding: this research received no external funding

Supplementary Table S1. Search strategies for PubMed database

PubMed
((((("temporomandibular joint"[MeSH Terms] OR ("temporomandibular"[All Fields] AND "joint"[All Fields]) OR "temporomandibular joint"[All Fields] OR ("temporomandibular joint"[MeSH Terms] OR ("temporomandibular"[All Fields] AND "joint"[All Fields]) OR "temporomandibular joint"[All Fields] OR "tmj"[All Fields])) AND ("disc"[All Fields] AND ("displace"[All Fields] OR "displaced"[All Fields] OR "displacement, psychological"[MeSH Terms] OR ("displacement"[All Fields] AND "psychological"[All Fields]) OR "psychological displacement"[All Fields] OR "displacement"[All Fields] OR "displacements"[All Fields] OR "displaces"[All Fields] OR "displacing"[All Fields]))) OR ("disk"[All Fields] AND ("displace"[All Fields] OR "displaced"[All Fields] OR "displacement, psychological"[MeSH Terms] OR ("displacement"[All Fields] AND "psychological"[All Fields]) OR "psychological displacement"[All Fields] OR "displacement"[All Fields] OR "displacements"[All Fields] OR "displaces"[All Fields] OR "displacing"[All Fields]))) AND ("facial asymmetry"[MeSH Terms] OR ("facial"[All Fields] AND "asymmetry"[All Fields]) OR "facial asymmetry"[All Fields])) OR (("functional laterality"[MeSH Terms] OR ("functional"[All Fields] AND "laterality"[All Fields]) OR "functional laterality"[All Fields] OR "laterality"[All Fields] OR "lateral"[All Fields] OR "lateralisation"[All Fields] OR "lateralisations"[All Fields] OR "lateralise"[All Fields] OR "lateralised"[All Fields] OR "lateralises"[All Fields] OR "lateralising"[All Fields] OR "lateralities"[All Fields] OR "lateralization"[All Fields] OR "lateralizations"[All Fields] OR "lateralize"[All Fields] OR "lateralized"[All Fields] OR "lateralizes"[All Fields] OR "lateralizing"[All Fields] OR "laterally"[All Fields] OR "laterals"[All Fields]) AND ("mandible"[MeSH Terms] OR "mandible"[All Fields] OR "mandibles"[All Fields] OR "mandible s"[All Fields]) AND ("shift"[All Fields] OR "shifted"[All Fields] OR "shifting"[All Fields] OR "shiftings"[All Fields] OR "shifts"[All Fields])) OR (("functional laterality"[MeSH Terms] OR ("functional"[All Fields] AND "laterality"[All Fields]) OR "functional laterality"[All Fields] OR "laterality"[All Fields] OR "lateral"[All Fields] OR "lateralisation"[All Fields] OR "lateralisations"[All Fields] OR "lateralise"[All Fields] OR "lateralised"[All Fields] OR "lateralises"[All Fields] OR "lateralising"[All Fields] OR "lateralities"[All Fields] OR "lateralization"[All Fields] OR "lateralizations"[All Fields] OR "lateralize"[All Fields] OR "lateralized"[All Fields] OR "lateralizes"[All Fields] OR "lateralizing"[All Fields] OR "laterally"[All Fields] OR "laterals"[All Fields]) AND ("mandible"[MeSH Terms] OR "mandible"[All Fields] OR "mandibular"[All Fields] OR "mandibulars"[All Fields]) AND ("shift"[All Fields] OR "shifted"[All Fields] OR "shifting"[All Fields] OR "shiftings"[All Fields] OR "shifts"[All Fields])) OR (("functional laterality"[MeSH Terms] OR ("functional"[All Fields] AND "laterality"[All Fields]) OR "functional laterality"[All Fields] OR "laterality"[All Fields] OR "lateral"[All Fields] OR "lateralisation"[All Fields] OR "lateralisations"[All Fields] OR "lateralise"[All Fields] OR "lateralised"[All Fields] OR "lateralises"[All Fields] OR "lateralising"[All Fields] OR "lateralities"[All Fields] OR "lateralization"[All Fields] OR "lateralizations"[All Fields] OR "lateralize"[All Fields] OR "lateralized"[All Fields] OR "lateralizes"[All Fields] OR "lateralizing"[All Fields] OR "laterally"[All Fields] OR "laterals"[All Fields]) AND ("mandible"[MeSH Terms] OR "mandible"[All Fields] OR "mandibles"[All Fields]

OR "mandible s"[All Fields]) AND ("deviate"[All Fields] OR "deviated"[All Fields] OR "deviates"[All Fields] OR "deviating"[All Fields] OR "deviation"[All Fields] OR "deviational"[All Fields] OR "deviations"[All Fields])) OR (("functional laterality"[MeSH Terms] OR ("functional"[All Fields] AND "laterality"[All Fields]) OR "functional laterality"[All Fields] OR "laterality"[All Fields] OR "lateral"[All Fields] OR "lateralisation"[All Fields] OR "lateralisations"[All Fields] OR "lateralise"[All Fields] OR "lateralised"[All Fields] OR "lateralises"[All Fields] OR "lateralising"[All Fields] OR "lateralities"[All Fields] OR "lateralization"[All Fields] OR "lateralizations"[All Fields] OR "lateralize"[All Fields] OR "lateralized"[All Fields] OR "lateralizes"[All Fields] OR "lateralizing"[All Fields] OR "laterally"[All Fields] OR "laterals"[All Fields]) AND ("mandible"[MeSH Terms] OR "mandible"[All Fields] OR "mandibular"[All Fields] OR "mandibulars"[All Fields]) AND ("deviate"[All Fields] OR "deviated"[All Fields] OR "deviates"[All Fields] OR "deviating"[All Fields] OR "deviation"[All Fields] OR "deviational"[All Fields] OR "deviations"[All Fields]))) AND ("child"[MeSH Terms] OR "child"[All Fields] OR "children"[All Fields] OR "child s"[All Fields] OR "children s"[All Fields] OR "childrens"[All Fields] OR "childs"[All Fields])

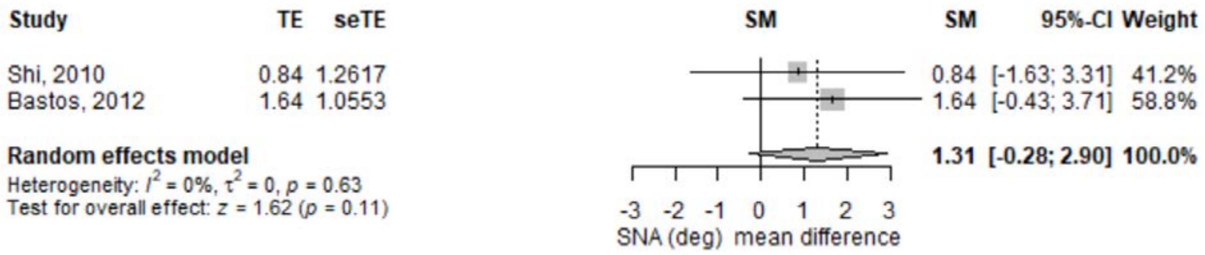

Supplementary Figure S1 . Forest plot for (deg) standardized mean change difference. SNA-sella nasion point A angle, TE - effect; seTE - the standard error of the effect; SM - mean difference; CI - confidence interval.

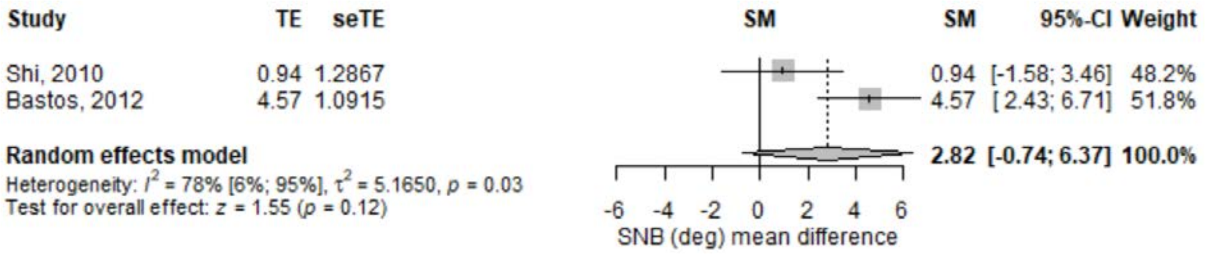

Supplementary Figure S2. Forest plot for (deg) standardized mean change difference. SNB-sella nasion point B angle, TE - effect; seTE - the standard error of the effect; SM - mean difference; CI - confidence interval.

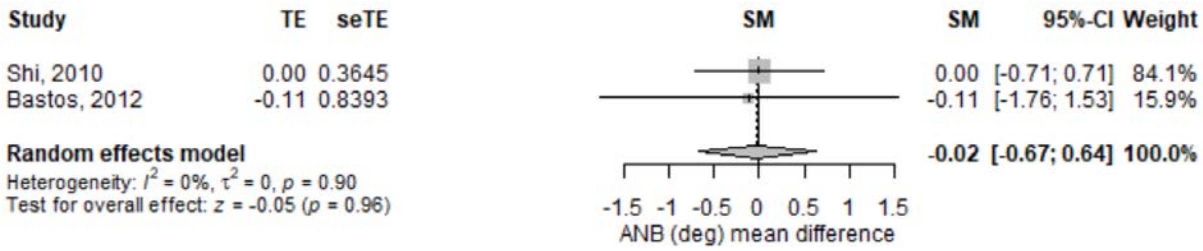

Supplementary Figure S3. Forest plot for (deg) standardized mean change difference. ANB-point A – nasion - point B angle, TE - effect; seTE - the standard error of the effect; SM - mean difference; CI - confidence interval.

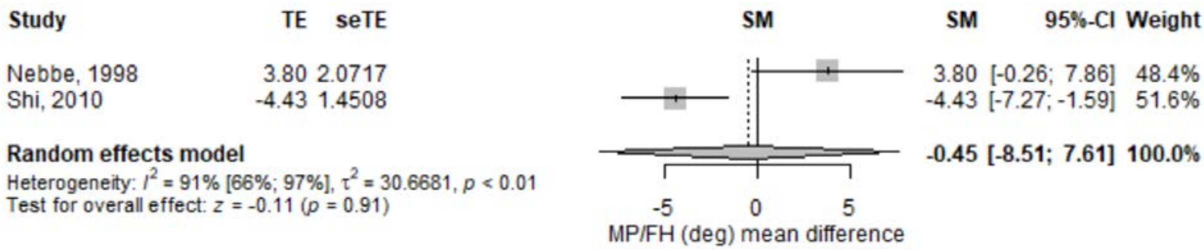

Supplementary Figure S4. Forest plot for (deg) standardized mean change difference. MP-mandibular plane, FM-Frankfurt plane, TE - effect; seTE - the standard error of the effect; SM - mean difference; CI - confidence interval.

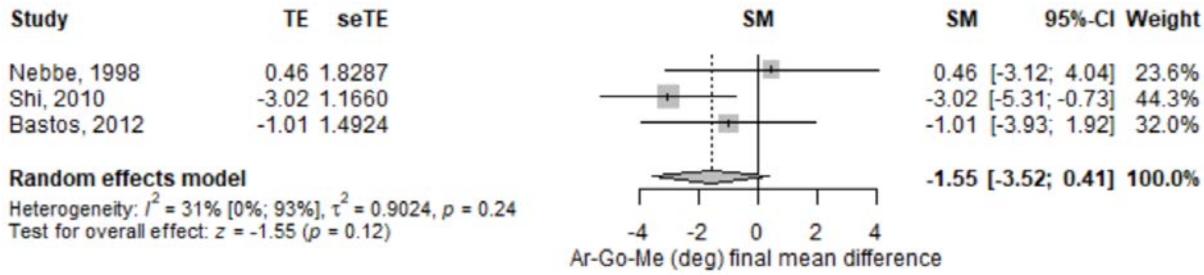

Supplementary Figure S5. Forest plot for (deg) standardized mean change difference. Ar-articulare, Go-gonion, Me-menton, TE - effect; seTE - the standard error of the effect; SM - mean difference; CI - confidence interval.

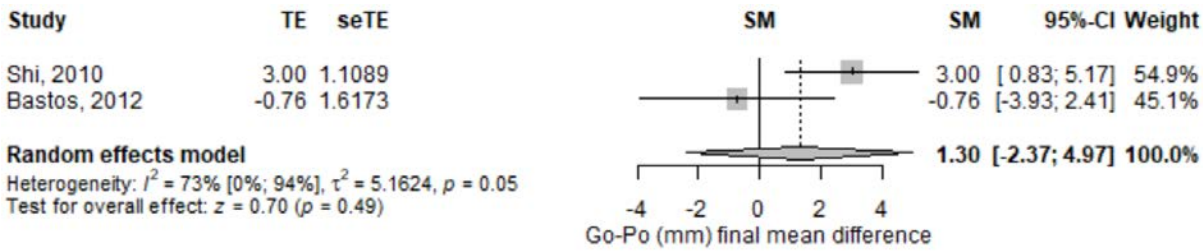

Supplementary Figure S6. Forest plot for (mm) standardized mean change difference. Go-gonion, Po=porion, TE - effect; seTE - the standard error of the effect; SM - mean difference; CI - confidence interval.

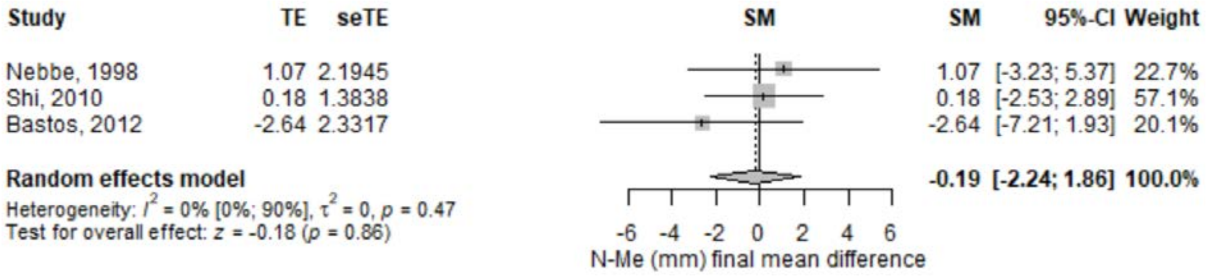

Supplementary Figure S7. Forest plot for (mm) standardized mean change difference. TE - effect; seTE - the standard error of the effect; SM - mean difference; CI - confidence interval.

Sensitivity analysis

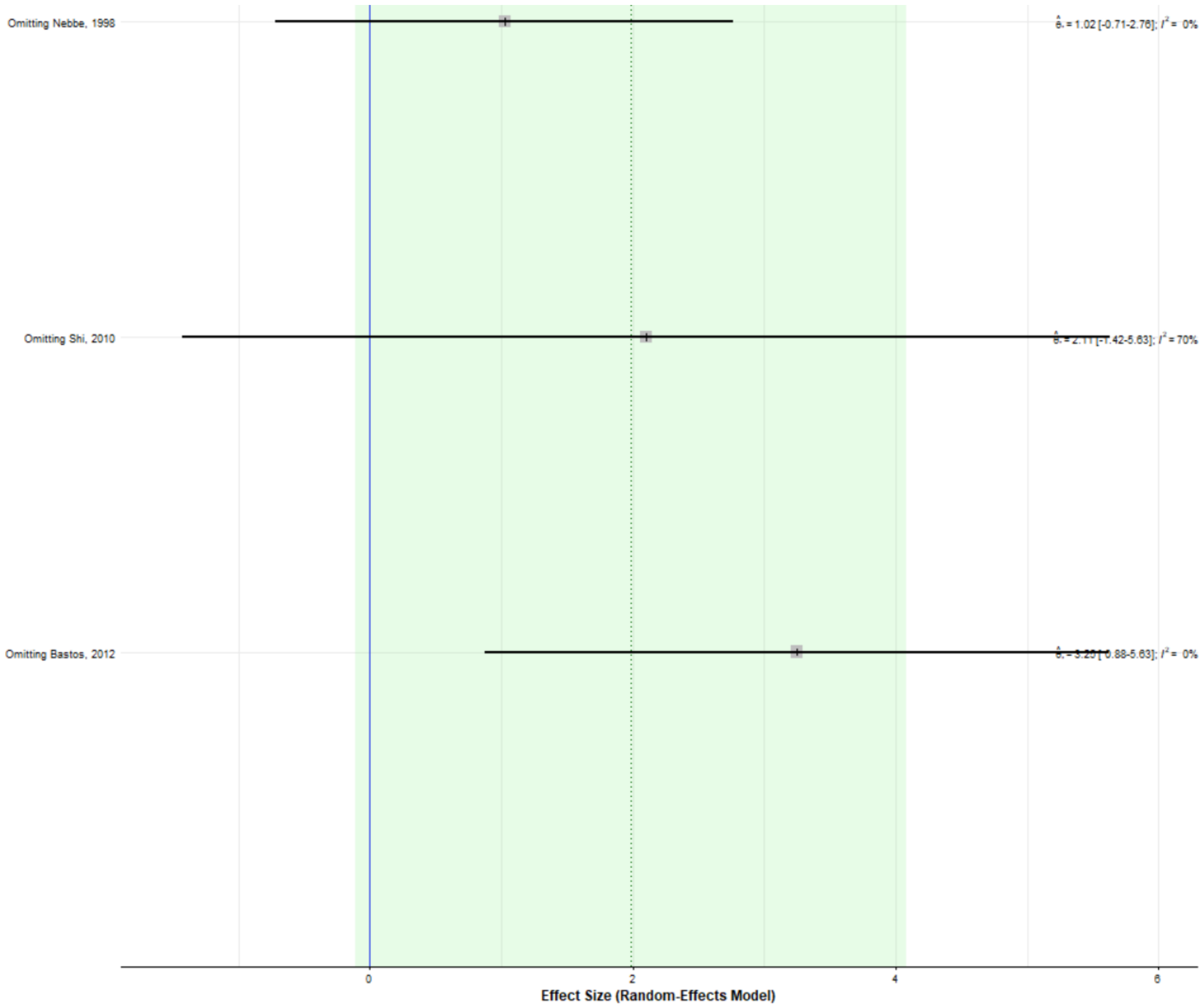

Supplementary Figure S8. Leave-one-out sensitivity analysis for articulare to gonion distance

$\hat{\Theta}$ = mean difference

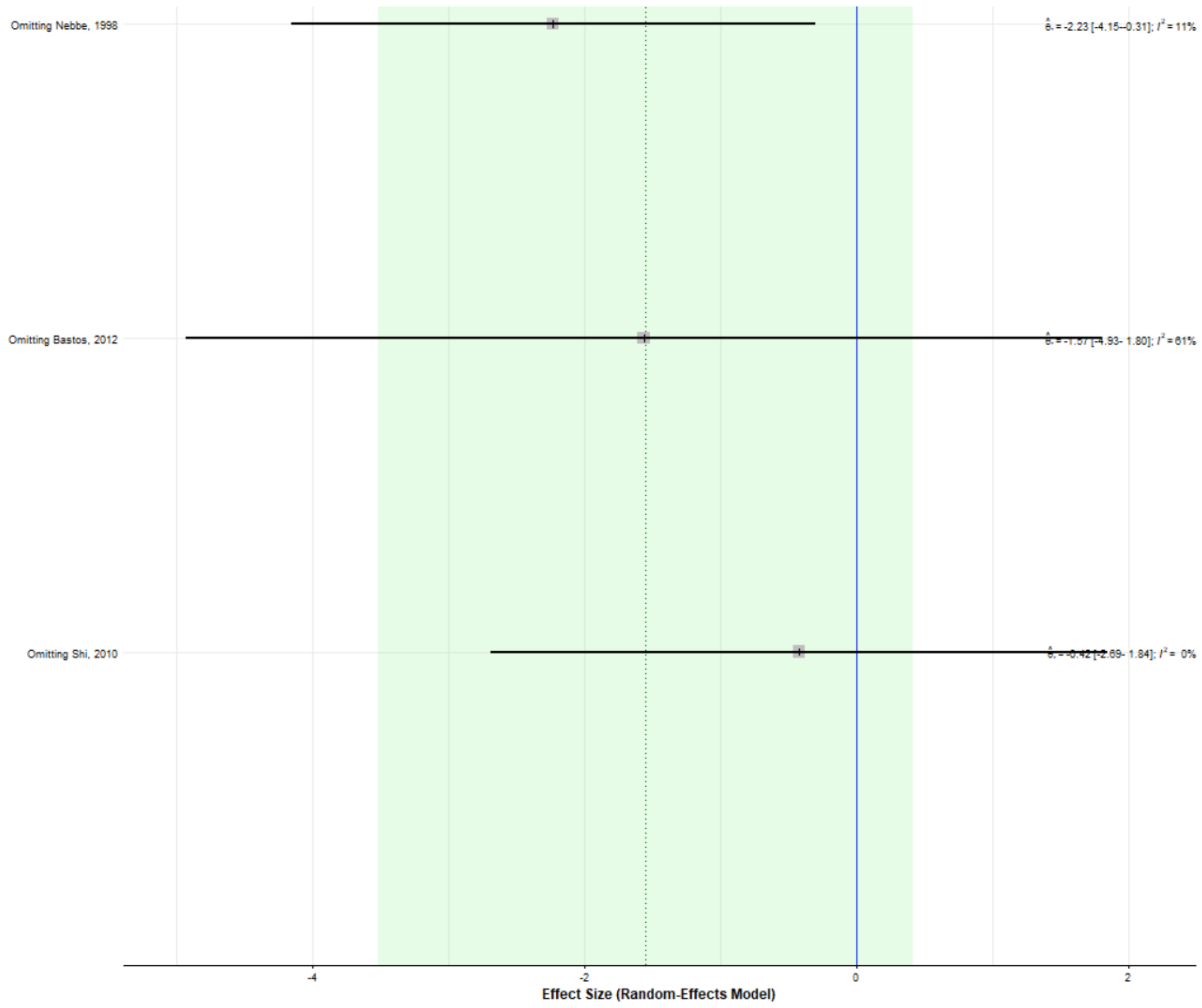

Supplementary Figure S9. Leave-one-out sensitivity analysis for Ar-Go-Me

$\hat{\Theta}$ = mean difference, Ar-articulare, Go-gonion, Me-menton

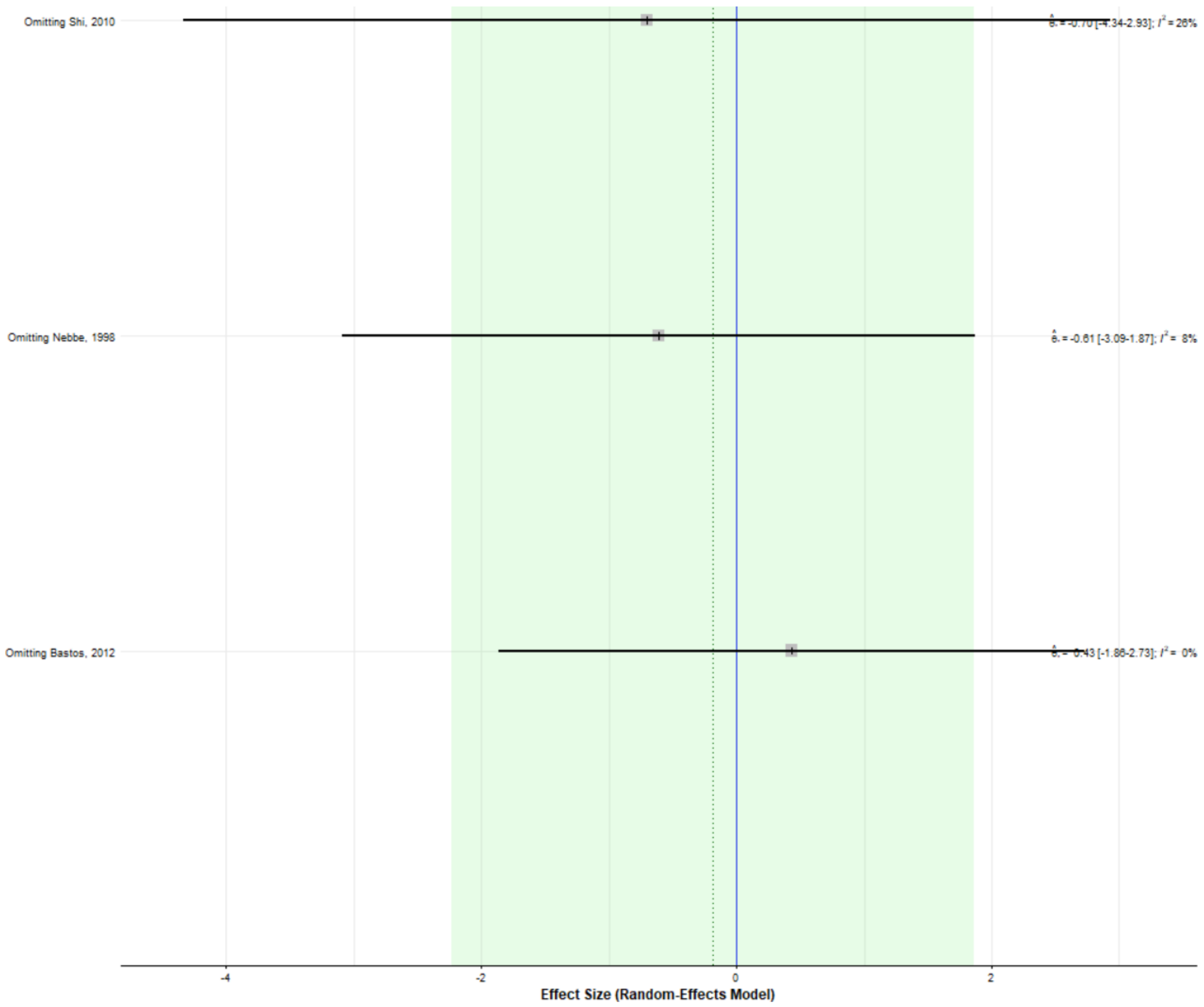

Supplementary Figure S10. Leave-one-out sensitivity analysis for N-Me

$\hat{\Theta}$ = mean difference, N-nasion, Me-menton
